# Signatures of Polaron Dynamics in Photoexcited MAPbBr_3_ by Infrared Spectroscopy

**DOI:** 10.1021/acs.jpcc.3c03668

**Published:** 2023-11-03

**Authors:** Valentina Carpenella, Claudia Fasolato, Diego Di Girolamo, Jessica Barichello, Fabio Matteocci, Caterina Petrillo, Danilo Dini, Alessandro Nucara

**Affiliations:** † Department of Sciences, University of Roma Tre, Via della Vasca Navale 84, 00146 Rome, Italy; ‡ CNR-ISC, Istituto dei Sistemi Complessi, c/o Sapienza University of Rome, P.le A. Moro 5, 00185 Rome, Italy; § Department of Chemistry, Sapienza University of Rome, P.le A. Moro 5, 00185 Rome, Italy; ∥ CHOSE, Department of Electronic Engineering, University of Rome Tor Vergata, Rome 00133 Italy; ⊥ Department of Physics and Geology, University of Perugia, Via A. Pascoli, 06123 Perugia, Italy; # CNR-SPIN and Department of Physics, Sapienza University of Rome, Piazzale Aldo Moro 5, 00185 Rome, Italy

## Abstract

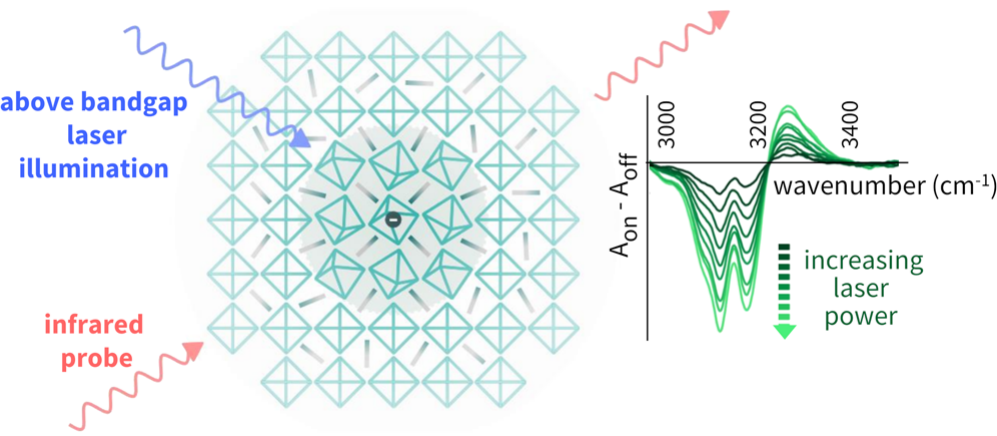

Hybrid organic–inorganic perovskites (HOIPs) have attracted considerable attention in the past years as photoactive materials for low-cost, high-performance photovoltaics. Polaron formation through electron–phonon coupling has been recognized as the leading mechanism governing charge carrier transport and recombination in HOIPs. In this work, two types of MAPbBr_3_ film samples deposited on different substrates (transparent insulating SrTiO_3_ and a heterostructure mimicking a functioning photovoltaic cell) were photoexcited with above-bandgap radiation at 450 nm, and the effects of illumination on the sample were analyzed in the infrared region. The infrared absorbance detected at different powers of the photoexciting laser allowed us to obtain an estimate of the characteristic decay time of photoexcited polaron population of the order of 100–1000 ns. When focusing on the absorption features of the MA molecular cation in the region of the NH stretching modes, we observed the influence of hydrogen bonding and the effect of the polaron dynamics on the cation reorientation.

## Introduction

Hybrid organic–inorganic perovskites (HOIPs) have been intensively studied in the past few decades owing to their remarkable performances in photovoltaic applications. The interest in these materials lies in their unique characteristics: direct bandgap tunable in the visible spectrum,^1^ long-lived charge carriers with modest recombination rates, and long carrier diffusion lengths.^2−4^ The general formula of HOIPs is ABX_3_, in which B is a divalent metal cation (namely, Pb^2+^ or Sn^2+^), X is a halogen anion (I^–^, Br^–^, or Cl^–^) arranged in a inorganic framework of corner-sharing octahedrons, and A is a molecular cation, usually a methylammonium (of chemical formula CH_3_NH_3_^+^ or MA) or a formamidinium (HC(NH_2_)_2_^+^, FA), located within the voids of the inorganic lattice. The inorganic scaffold is rather soft, as already assessed by some high-pressure studies on this class of compounds,^5,6^ and thus can be easily distorted through the effect of a traveling charge. It was indeed verified that, in HOIPs, the photogenerated charge carriers can couple with the polarization field of the distorted lattice, giving rise to large polarons.^7−11^ The photoinduced polaron formation has been the subject of numerous studies which confirmed the Fröhlich-like nature of these quasi-particles, *i.e.*, a deformation with large spatial extension and small effective mass.^11−14^ Positive (from holes) and negative (from electrons) polarons, in fact, generate different local deformations of the lattice, which involve up to 10 unit cells, and have comparable binding energies of the order of 0.2 eV.^15^ The phenomenon of self-trapping and the consequent small mobility are typical features of these quasi-particles, which have been proposed as the main factors influencing carrier recombination.^15^

In this framework, the dynamics of the monovalent cation A plays a central role, being itself affected by the local lattice distortion, while influencing the carriers’ mobility, thanks to its orientational degrees of freedom.^16,17^ All of the experimental data and calculation hereto available agree that the MA orientation is highly influenced by the potential energy landscape induced by the lattice deformations and by thermal energy.^18,19^ In the cubic phase of these perovskites (space group *Pm*3*m*), MA can reorient almost as a freely rotating molecule on eight energetically degenerate orientations at the center of the cubic cell. The rate of reorientation of the cation lies in the picosecond time scale (14 ps at room temperature), and the hopping energy is well below the room-thermal activation.^18^ It has been predicted that an electronic charge carrier can prompt an orientational rearrangement of the organic dipoles while crossing the crystalline lattice.^7,20^ The reorientation of the MA molecule is thought to be one of the causes of charge screening, with consequent stabilization of the photogenerated carrier and prevention of fast recombination.^20^

The influence of the photoexcited polarons directly on the organic cation has been investigated so far only in a few cases. It has been commonly accepted that the cation A stabilizes and balances the spatial extent of positive and negative polarons, which, in turn, may exhibit optically active internal levels.^21−23^

The detection of photoinduced infrared-activated vibrations (IRAVs) has already been addressed as an effective way to attain evidence of polaron formation and to evaluate the importance of carrier–phonon interaction. Indeed, the IRAV peaks are observed in the absorption spectrum of a photoexcited system as a consequence of the lattice rearrangement in the excited electronic state, modifying the vibrational spectrum with relaxation of the IR selection rules, as observed in conjugated polymers and polar crystals.^24,25^ An extensive investigation was provided for MAPbI_3_.^15,21,26^ Experimental evidence demonstrates that both in the ultrafast pump–probe scheme, as well as in the out-of-equilibrium spectroscopy regime obtained by continuous wave (CW) illumination, IRAV features can be regarded as direct evidence of polaron formation in the early stage of the charge relaxation process. In principle, under the same conditions, IRAV modes might provide access to the role of the molecular cations involved in polaronic excitation. It is known that the A cations, caged in the deformed lattice, may undergo both an increase in the strength of the Br–HN hydrogen bonds and a reorientation to nonenergetically degenerate configurational states, in a similar way to what is observed in the cubic-tetragonal phase transition.^19^

In this work, we investigate both the photoinduced polaron and the MA vibrational spectrum of MAPbBr_3_. Compared to MAPbI_3_, MAPbBr_3_ has so far received less attention, and little is known about its energy spectrum under photoexcitation. This disparity is mainly prompted by the lower energy gap (1.4 eV) of the iodine-based HOIP, which is an appealing property for photovoltaic use. However, despite a higher gap value (∼2.3 eV), the bromide-based perovskite is less affected by moisture, oxygen, and heat under illumination;^27^ hence, it is suitable for the use over longer times. Moreover, high-bandgap Br-based perovskites possess the peculiarity of being colorful and semitransparent in the visible band, therefore ideal candidates for building integrated photovoltaics in windows and façades.^28^

We focus on the mid-infrared (MIR) spectrum of MAPbBr_3_ films deposited on different substrates. Our aim is to point out the close correlation between the steady polaron population that originated under illumination and the orientational and vibrational state of the MA molecular cations. Therefore, we first analyze in detail the spectral signatures of the large polarons formed under CW irradiation. Afterward, we focus on the vibrational spectrum of the MA cation in order to reveal the IRAV modes of the organic molecule and correlate them to the photoexcited polaron features. We observe the presence of the polaronic contribution as a broad band in the differential absorbance spectrum centered at the lowest frequencies of the MIR spectrum. Moreover, we detected new vibrational absorptions of the MA cations in correspondence with the modes mainly involved in the hydrogen bond network. We propose that the latter may be related to the different orientational states of the MA cations within the polaron-deformed lattice regions.

## Materials and Methods

### Sample Synthesis

The MAPbBr_3_ films were grown on two different substrates. One of the samples (hereafter sample A) was obtained using an FTO-coated glass bulk substrate on which a heterostructure formed by NiO–Al_2_O_3_–PbBr_2_ was previously deposited. NiO was deposited by spin coating of a 0.12 M NiCl_2_ solution in 2-methoxyethanol with 10 μL/ml HNO_3_ as a solution stabilizer. Annealing at 300 °C for 1 h results in a crystalline NiO film.^29^ The mesoporous Al_2_O_3_ layer was deposited from a nanoparticle dispersion, starting from Sigma-Aldrich 30% weight dispersion in isopropyl alcohol (IPA) and further diluting with IPA up to 1:3. After spin coating, the film is dried at 150 °C for 10 min. Dimetilformammide (DMF) solution containing PbBr_2_ was deposited by spin coating on top of the heterostructure. The film was subsequently heated for 10 min at 90 °C in a controlled environment and then submerged in a 15 mg/mL MABr solution in IPA, where the conversion to perovskite occurred within 5–10 min. Afterward, the perovskite film surface was rinsed with IPA and annealed at 150 °C for 10 min. The quality of the sample was tested by Raman spectroscopy (see SI Section S1 for further information). For the second sample (type B), a solution of molarity 1.4 M MABr and PbBr_2_ dissolved in dimethyl sulfoxide (DMSO) was prepared and stirred in a nitrogen-filled glovebox for 12 h. The 0.5 mm thick SrTiO_3_ (STO) substrate was exposed to a UV lamp for 30 min to improve the surface wettability and thus the perovskite deposition. After that, the STO substrate was heated up to 60 °C. The solution was later spun on the substrate and, after 10 s, 200 μL of ethyl acetate were dropped on the sample. Finally, the sample was sintered at 80 °C for 10 min. The thickness of the film was estimated acquiring the absorbance of the sample in three different sites of the surface on an optical benchtop setup. The three measurements, differing by less than 10%, were then averaged, and the mean thickness was calculated from the transmission value at the bandgap edge, using the conventional attenuation relation *T* = *T*_0_ exp(−μ·*d*), where μ is the attenuation coefficient taken from the literature (∼10^5^ cm^–1^, from ref (30)) and *d* is the sample thickness. The thickness of the perovskite film was estimated to be ∼300 nm.

The chemical degradation of the perovskite film of sample A was monitored by Raman spectroscopy. In the Raman spectra, except for an overall small decrease in the peak intensities (<20%), no new spectral features could be detected, proving that no additional chemical products have been created by environmental degradation. The physical degradation of the film, as monitored by optical microscopy, was also negligible. On sample B, only a moderate physical degradation was observed, consisting in the formation of MAPbBr_3_ microcrystals on the film surface, as revealed by optical microscopy. Nevertheless, the presence of crystalline microdomains on the sample did not interfere with the interpretation of our results. Both sample A and sample B were stored in the dark and in nitrogen-filled vials to avoid degradation.

### Infrared Measurements

The MIR experiments on both A and B films were performed with a commercially available Bruker IFS 66v/S Fourier transform infrared (FTIR) spectrometer equipped with a Hyperion microscope working in either transmission or reflection geometry. Spectra were recorded with 2 cm^–1^ resolution coadding 64 interferograms. Sample A was measured in reflectance mode using a gold slab as a reference, as the underlying heterostructure and the glass bulk both prevent the transmission of infrared radiation. The reflectance spectra, acquired before and during photoexcitation, were collected at normal incidence through the 15× microscope objective. Samples of type B were instead measured only in transmission mode, the STO substrate being transparent above 1000 cm^–1^, in the region of the MA vibrational modes. Algorithms from OPUS software^31^ were employed for integration and atmospheric vapor and baseline correction. Both reflectance and transmittance spectra provided information on the sample absorbance *A*(ω) since the latter can be obtained from reflectance spectra *R*(ω) exploiting the usual approximation for an infinite medium, *A*(ω) ∼ log (1/*R*(ω)).

Both *A* and *B* samples were photoexcited using a CW “pump” laser (Thorlabs PL450B laser diode, λ = 450 nm) with above-bandgap energy, focused on the film at a 45° tilt angle. The laser spot size was adjusted to match with the infrared one at the sample surface (spot size 400 × 900 μm^2^). To avoid heating and damaging of the samples during illumination, many (50) spectra of short duration (∼20 s) alternating on and off laser conditions were recorded and averaged. With the same approximation for an infinite medium, the differential absorbance Δ*A* can be evaluated directly from the ratio between the diffused reflectance collected with (*R*_*d*_^on^ (ω)) and without (*R*_*d*_^off^ (ω)) laser excitation .

### UV–Vis Measurements

UV–vis spectra were acquired on a Jasco V-570 spectrophotometer supplied with an ISN-470 integrating sphere attachment and a solid sample holder accessory. The UV–vis spectra of the perovskite films were obtained by comparing the diffused reflectance of the measured samples with that of a standard reference reflection plate (Spectralon).

## Results and Discussion

**Figure 1:**
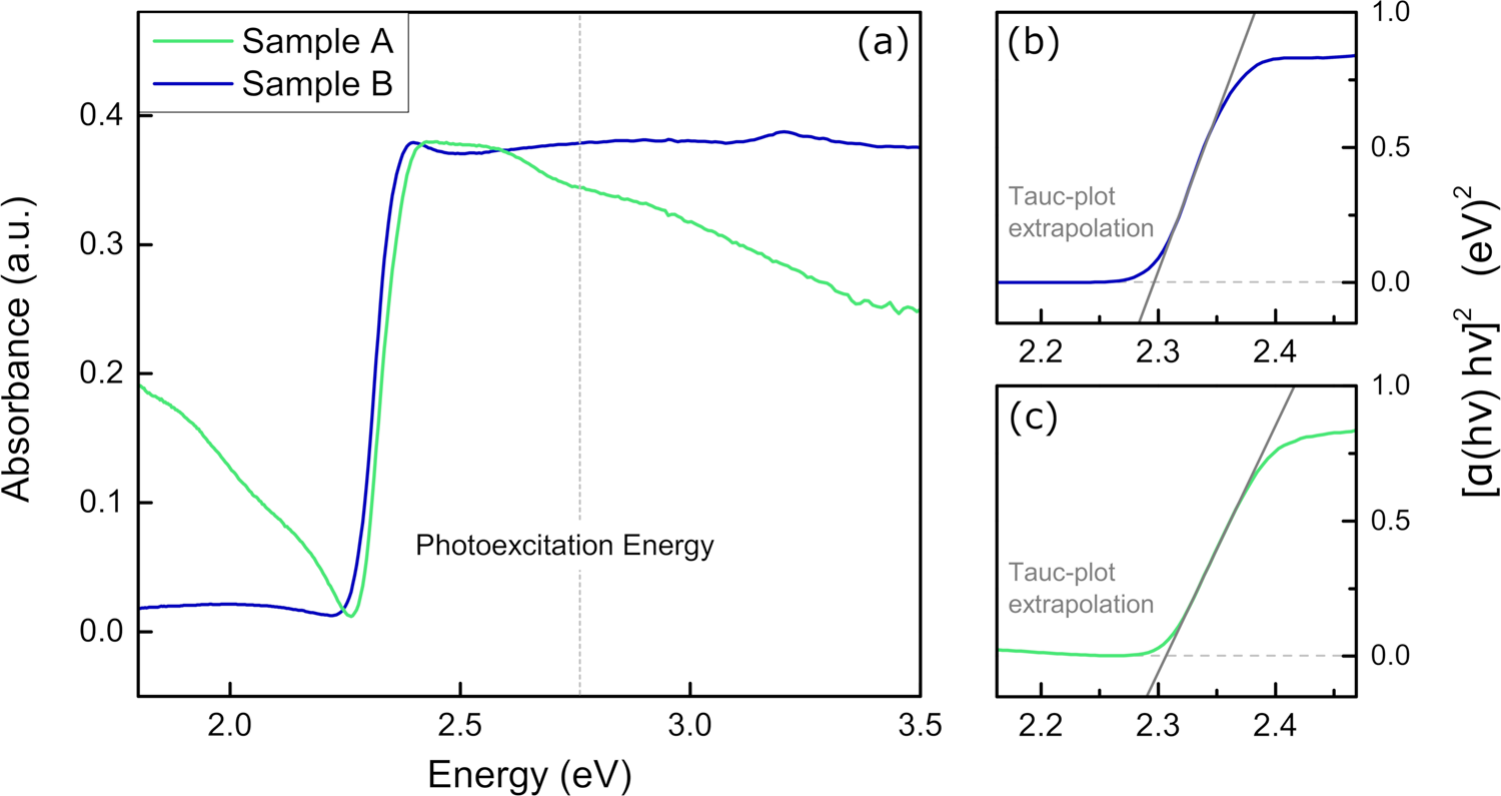
(a) Absorbance spectra in the UV–vis range of the MAPbBr_3_ film of samples A (in green) and B (in blue). (b, c) Tauc plot and linear extrapolation of the energy gap of sample B and sample A, respectively.

A preliminary investigation of the UV–vis absorbance of the selected HOIP films was performed to characterize our samples. The absorbance spectra of sample A (in green) and sample B (in blue) are shown in Figure 1a. The absorption edge of MAPbBr_3_ is well evident in both samples, and minor peaks in the spectrum of sample B are observed above the gap, in agreement with those reported by Hirasawa et al.^32^ The spectrum of sample A shows a general downward trend with energy (see Figure 1a) both below and above the absorption edge: this behavior is to be ascribed to the presence of the heterostructured substrate whose absorption in this range drives the total absorption observed from the whole sample (film + substrate). For more details on the substrate absorption spectrum, see SI Section 2. The analysis of the Tauc plots for both samples (Figure 1b,c) yields a bandgap value for MAPbBr_3_ of 2.30 ± 0.01 eV, consistent with that obtained from the photoluminescence spectrum (Figure S2 in the SI) and commonly reported in the literature.^30^ In this scenario, we chose to prompt above-bandgap absorption exciting the samples with laser radiation at 2.75 eV (wavelength λ= 450 nm).

**Figure 2:**
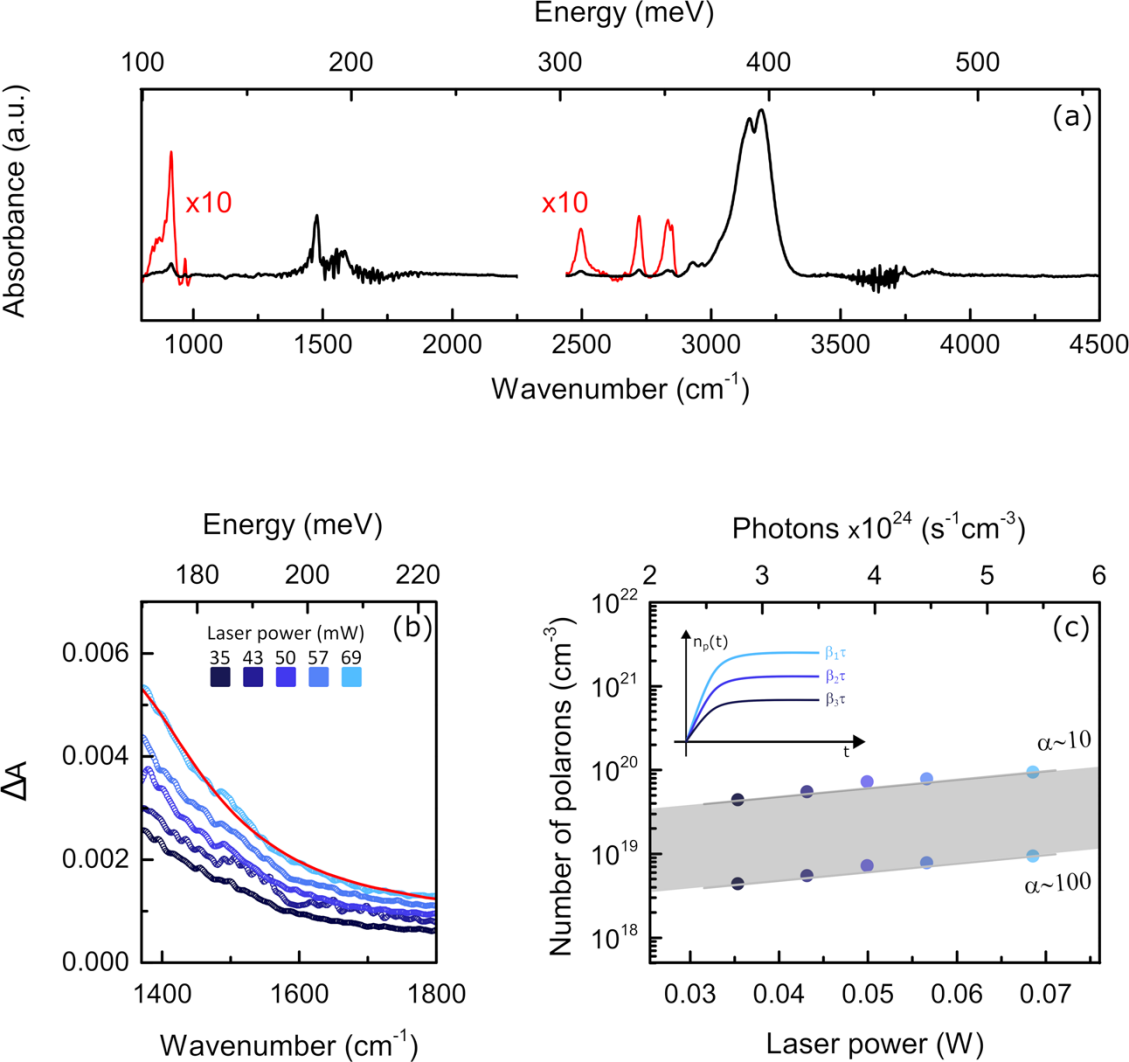
(a) Infrared absorbance spectrum of MAPbBr_3_ obtained from sample A. (b) Differential absorbance (see text) of sample B below 1800 cm^–1^. The red continuous line represents the Lorentzian fit to the polaron absorption feature. (c) Polaron density, estimated as explained in the text, as a function of laser power and incident photon density. The gray area highlights the uncertainty in the estimated polaron density values. Inset: schematic representation of polaron population over time at three different laser powers.

Figure 2a shows the IR absorbance of the unperturbed MAPbBr_3_ film obtained from a measurement of the reflectance of sample A. All of the infrared-active modes of the methylammonium molecule are clearly visible in the spectrum, and their energies are in good agreement with a previous study by Glaser et al.^33^ The relative intensity of the bands does not match the one retrieved in transmission measurements: this is expected in the diffuse reflectivity approach since, as in this case, wavelength dependence of the scattering from grain boundaries affects the spectral shape.^34^

The lowest frequency vibrational modes at 917 and at 968 cm^–1^ are assigned to the CH_3_–NH_3_^+^ rocking motion and to the C–N stretching, respectively. The absorption bands at 1478 and 1582 cm^–1^ correspond to the symmetric and asymmetric NH_3_^+^ bending. At higher energies, the weak absorptions at 2497, 2721, and 2834 cm^–1^ arise from the combination of vibrational modes. Finally, the asymmetric stretching of CH_3_ at 2963 cm^–1^ and the symmetric and asymmetric stretching of NH_3_^+^ at 3149 and 3195 cm^–1^, respectively, are well recognized in the spectrum.

In Figure 2b, we show the absorbance variation Δ*A* = *A*_on_ – *A*_off_ of sample B in the region below 1800 cm^–1^, observed in the presence of above-bandgap photoexcitation with increasing power of the external laser. In line with previous observations in MAPbI_3_,^15,21^ we ascribe these spectral changes to polaronic effects and, more precisely, to the photoionization process of a large polaron. Previous data in the literature reported polaron photoionization energies of 190 meV for MAPbI_3_^15^ and 160 meV for CsPbBr_3_.^7^ A Lorentzian fit to our data, shown in Figure 2b, provides a peak energy of 1315 cm^–1^ (163 meV), in good agreement with previous observations. For HOIPs, an intermediate electron–phonon coupling strength (α_el-ph_ ∼ 1.6–1.8) is predicted in the literature;^10,35,36^ thus, the polaron photoionization spectral contribution should extend somewhat above the longitudinal phonon spectral region (<200 cm^–1^). However, for MAPbI_3_, this band has been observed between 1200 and 1500 cm^–1^,^15,21^ indicating that a stronger coupling regime occurs. Owing to the limited spectral range attainable in our measurements, we cannot confirm such an assumption, but the presence of a polaron band extending to the MIR might suggest polaron pinning in these compounds.

The intensity of Δ*A* shows a marked dependence on the laser power (Figure 2c). It is physically sound to assume that CW illumination generates a steady population of polarons, as illustrated in the inset of Figure 2c, whose value is *n*_p_ = τβ, with β being the polaron photoinduction rate and τ the characteristic lifetime of the polaron population (see SI Section 3 for further details). According to ref (37), *n*_p_ can be independently estimated from the differential absorbance through the relationship *n*_p_ ≈ Δ*A*/σ*d*, where σ is the absorption cross section in the region of the polaronic absorption and *d* is the film thickness (*d* ∼ 300 nm). The evaluation of the cross section σ is not straightforward, but a reasonable assessment can be derived from the average polaron volume *V*_p_ (polaronic radius 4.2 nm, according to ref (10)) and the absorption coefficient α (cm^–1^) through the relation σ = *V*_p_·α. Unfortunately, α is affected by a large uncertainty, and its estimate provides values ranging from 10 to 100 cm^–1^ (see SI Section 4 for more details).

Therefore, within these approximations and making use of the values of Δ*A* obtained from fits to our data, we estimate a steady polaron population of the order of 10^18^–10^19^ polaron/cm^3^ in the illuminated region of the sample.

The top *x* axis in Figure 2c refers to the number of photons *n* delivered by the laser (see Section 5 of the SI): the rate of photoinduced polarons (β) will be proportional to the photon flux, β = ε*n*, where ε indicates the photon–polaron conversion efficiency. Since ε is unknown, we cannot unambiguously estimate the polaron population lifetime τ by the slope of the data in Figure 2c, but lower limits for this quantity lie in the interval between 100 and 1000 ns when assuming maximum efficiency (ε = 1).

Values for polaron formation time and polaron lifetime reported in the literature span several orders of magnitude, from picoseconds to milliseconds, depending on the incident energy time- and space-density, sample composition, and experimental setup. In Table 1, we report a selection of previous observations of polaron lifetime in HOIPs indicating, for each, the experimental technique employed.

**Table 1:** Reports of the Polaron Lifetime from the Literature for Different HOIP Compounds

sample	polaron lifetime τ	experimental technique
MAPbI_3_^15^	∼1 ms	CW-photoexcited IR absorption spectroscopy (same as present work, but at *T* = 78 K)
MAPbI_3_^38^	∼140 ns	time-resolved photoluminescence and transient absorption spectroscopy
MAPbI_3_ (polycrystalline)^39^	30 μs	steady-state photoconductivity
MAPbBr_3_ (single crystal)^39^	∼3 ms	steady-state photoconductivity
MAPbBr_3_^40^	up to 18 μs	time-resolved photoluminescence and time-resolved infrared spectroscopy
MAPbBr_3_^41^	100 ns	two-photon photoluminescence spectroscopy
MAPbI_3-*x*_Cl_*x*_^3^	∼300 ns	ns transient absorption and time-resolved photoluminescence spectroscopy

In principle, a longer lifetime of the polaron population is expected when the constraints of the bottleneck effect are fulfilled.^42−44^ Indeed, the lowest decay times seem to be observed at the smallest carrier densities and in pump–probe experiments: the time scales of pump–probe optical approaches are much faster than the lattice relaxation dynamics, meaning that the incident photons probe the polarons over time scales shorter than their thermalization with the lattice, which is thus still “cold” at such short delays. Conversely, in CW experiments, the local heating of the sample and the slow detection procedures give access to the polaron–thermal bath interaction. Indeed, upon CW illumination, LA phonon states are also excited (we remark that a local temperature around 400 K was estimated in our experiment, as reported in Section 6 of the SI), so that the exceeding LA steady population enhances the phonon bottleneck effect, providing even longer decay times for polarons. It is worth noting that high concentrations of photocarriers (>10^18^) are also predicted to inhibit polaron stabilization in pump–probe regimes;^45,46^ however, an effective threshold is not available in the literature nor this effect has been predicted to exist in CW experiments, where the interaction of polarons with a warm lattice bath plays the key role.

**Figure 3:**
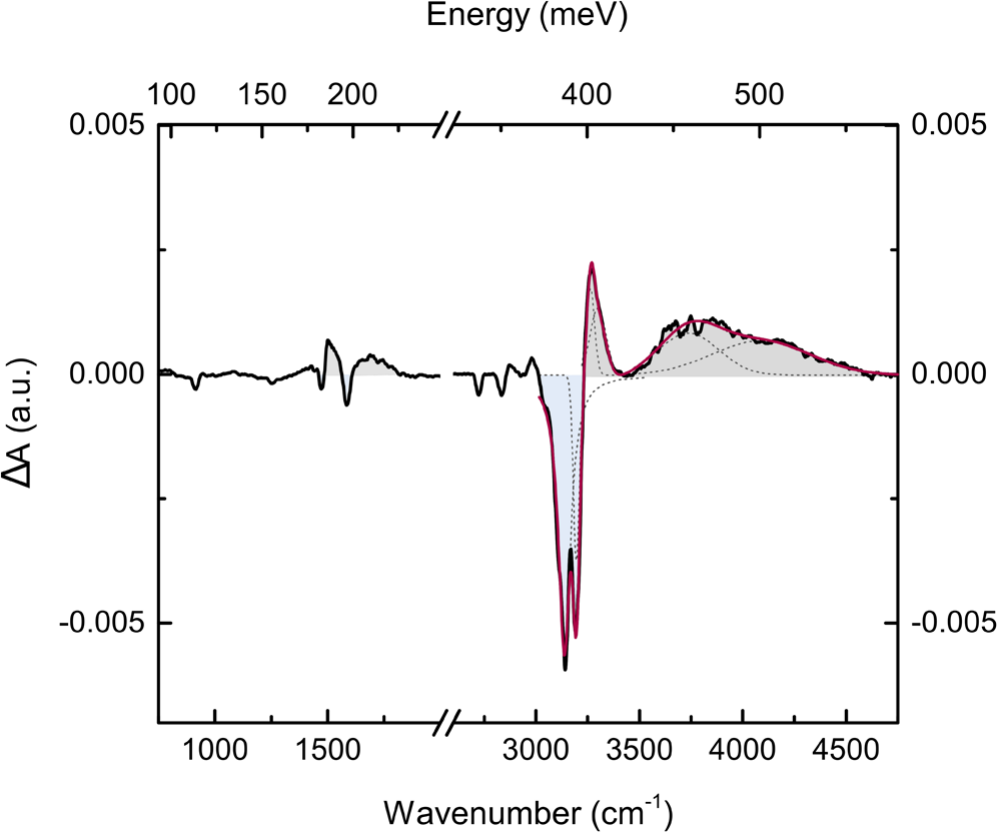
Variation of the absorbance spectrum of sample A observed upon continuous illumination with 450 nm, 69 mW laser photoexcitation. The quantity Δ*A* is defined as Δ*A* = *A*_on_ – *A*_off_ (see the text). Positive and negative contributions to the spectrum are highlighted, respectively, in gray and light blue. The Gaussian fitting deconvolution of the positive and negative contributions is indicated by the dashed lines.

The differential absorption Δ*A*, measured on sample *A* in the MIR region, is shown in Figure 3. At the main vibrational modes (see Figure 2a), the Δ*A* spectra exhibit both positive (gray areas in Figure 3) and negative (light-blue areas) features. The negative values of Δ*A* are in correspondence to the absorption peaks measured on the unperturbed sample, meaning that absorption is suppressed by the photoexcitation. These marked drops in the differential spectrum are accompanied by positive Δ*A* features at higher wavenumbers, which imply the appearance of new IR-active absorptions in the photoexcited film, close to the unperturbed vibrational modes. Such Δ*A* variations occur in each vibrational mode of MA but are much more evident for the intense NH stretching above 2900 cm^–1^. We remark that direct thermal effects for these vibrational energies can be definitely excluded, as thoroughly discussed in Section S6 of the SI.

As shown in Figure 3, fitting the negative region of the spectrum returns two modes centered at 3138 and 3195 cm^–1^, in correspondence, within the experimental error, to the NH_3_^+^ symmetric and asymmetric stretching, respectively; the two broad bands at positive Δ*A* are observed at higher wavenumbers, the first centered at 3281 cm^–1^, about 100 cm^–1^ from the central frequency of the unperturbed band, and the other, broader and less intense, with maximum at 3750 cm^–1^, shifted from the same band by ∼600 cm^–1^.

**Figure 4:**
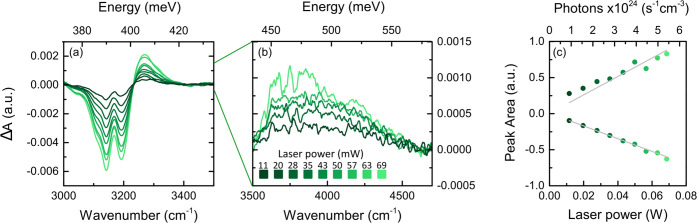
(a, b) Differential absorbance spectra of sample A at different output powers of the pumping laser beam in the range of 3000–3500 and 3500–4700 cm^–1^, respectively. Some data in panel (b) are omitted for the sake of clarity. The baseline subtraction performed on these spectra is discussed in Section S8 of the SI. (c) Calculated peak areas in the range of 3000–4500 cm^–1^ as a function of laser power and the number of photons impinging on the sample.

In the case of the MAPbBr_3_ film deposited on a bare SrTiO_3_ (sample B), the transmission measurements in the same NH stretching region highlighted only the most intense positive band (Figure S4 of the SI) due to the unfavorable signal/noise ratio of the setup; however, the features found in transmission experiments provide a further validation of the reflectivity experiments. We note that the differential absorbance from sample A (Figure 4a) is at least an order of magnitude higher than that of sample B (Figure S5 of SI, Section 7). However, we remark that experimental configurations adopted to measure samples A and B are quite different (diffuse reflectance for the former and direct transmission for the latter) so that physical parameters, such as sample thickness and diffusion efficiency, prevent a reliable quantitative comparison between the samples. Therefore, we can only infer qualitatively that MA vibrational changes occur in both samples.

An explicative magnification on the regions of interest at different laser power excitations is shown in Figure 4a,b. The area of the negative Δ*A* region and the sum of the two positive areas obtained from Sample A are shown as a function of pump power in Figure 4c. The enhancement of the negative area versus power reveals the progressive depopulation of the vibrational NH_3_^+^ ground states. It is indeed remarkable that the sum of the positive areas follows the same linear trend as that of the negative one, suggesting that the fraction of NH oscillators leaving the unperturbed vibrational ground state (negative area) strictly matches the increase in the population of new vibrational ground states (positive area).

The emergence of new vibrational modes in polar crystals is a well-established occurrence.^24,25,47^ It is well-known that IRAV modes originate from polarons since they arise in polar lattices as a result of the local deformations induced around charges and are indicative of the coupling between the charged excitation and the surrounding lattice.^25^ However, IRAVs signatures are expected primarily at low wavenumbers (below 500 cm^–1^), i.e., close to the absorption lines of the main phonons. The spectral features here observed in Δ*A* are IRAV modes ascribable to a different effect: our hypothesis is that the motion of the organic cation is heavily affected by the polaronic lattice deformation through the elongation and/or the shortening of the Br–H hydrogen bonds. We hypothesize that the steady polaron population formed upon irradiation distorts the inorganic lattice involving a significant percentage of the organic molecular cations (estimated at around 10% of the total MA cations). These lattice distortions could temporarily “freeze” the MA cations in specific orientations where they experience different hydrogen bond strengths with the surrounding Br^–^ ions. Indeed, as already discussed by Stallhofer et al. for MAPbI_3_,^21^ changes in the bond strength between atoms can result in a frequency shift of the IR modes. Recently, Lee and co-workers^19^ asserted that the 8-fold degenerate orientational states of the MA ion in the cubic *O_h_* symmetry are grouped into two manifolds of states when lattice changes into the tetragonal *D*_2*d*_ symmetry; this degeneracy breaking is a consequence of H bonding strength changes. In this scenario, the two positive bands observed in our Δ*A* spectra above 3200 cm^–1^ could be considered as the vibrational bands of MA ions frozen in two main orientational configurations within lattice regions distorted by the presence of polarons. As mentioned in ref (19), the energy gap between the two locked orientations in the *D*_2*d*_ symmetry phase (see Section S9 of the SI) is theoretically estimated at around 90 meV, thus in good agreement with the energy difference Δ*E* that we obtain from the peak energies of the observed bands. We cannot push the analogy with the ordered orientational phase *D*_2*d*_ any further, as the photoinduced distortion could produce local strains with different ion arrangements. A more detailed analysis of the photogenerated IRAV modes shows that each one of them is composed of two bands, and a qualitative deconvolution with Gaussian lines is shown in Figure 3. The presence of multiple contributions for each band can be related either to a slightly different lattice deformations due to positive (holes) and negative (electrons) polarons or to a further symmetry removal between the manifold of orientational states. The binding energy difference between positive and negative polarons in MAPbI_3_ is of the order of 0.05 eV,^15^ a value similar to the difference of the central energy of the two Gaussian components describing the IRAV vibrational mode at 3750 cm^–1^. As for the deconvolution of the IRAV feature at lower frequency (centered around 3250 cm^–1^), the Gaussian lines are separated only by 40 cm^–1^ (0.005 eV); therefore, a degeneration removal between orientational states appears as a more plausible hypothesis for such a fine structure.

## Conclusions

In this work, we studied the MIR spectrum of two samples of MAPbBr_3_: a heterostructure whose composition mimics a working photovoltaic cell and a perovskite film on SrTiO_3_ which, in the context of this work, was used to confirm the main results and to better highlight the spectral contributions in the polaronic regions. For the first time, to our knowledge, the contribution of polarons in the MIR as a function of the optical output power of the pump laser in MAPbBr_3_ was explored, thus linking the IRAV spectral modifications associated with the MA vibrational features to the estimated photoexcited polaron population. The experimental data confirm the long lifetime, 100–1000 ns, of this population of quasi-particles. Their excitation energies are found to be higher than those expected from the Fröhlich models of large polarons: the combination of the two outcomes suggests a picture of soft large polarons trapped in the lattice, similar to those observed in iodine-based HOIPs. The above-gap irradiation also affects the vibrational states of the molecular organic ions, with the rise of new absorption bands to the detriment of the population of the fundamental levels of the unperturbed states. Our hypothesis is that these IRAVs are related to modified orientational configurations of the methylammonium ions, following the deformation of the lattice produced by the polarons.

This work provides a solid experimental approach to the study of the orientational and vibrational dynamics of the organic cations of HOIPs and highlights the close correlation between the local lattice strains and the degrees of freedom of the organic molecules. The knowledge of the relevant coupling mechanisms between organic–inorganic lattices can provide indications of polaron photoexcitation in HOIPs that are not yet fully understood. The results presented here are relevant for the engineering of materials suitable for use in photovoltaics: indeed, the present approach could highlight the role of the organic cations in determining the lifetime and mobility of the excited charge states and suggest routes to tailor these properties.

## Supporting Information Available

Further details on the sample characterization and on the analysis of the differential absorbance spectra (PDF)
