# Supplementary material for: Signatures of Polaron Dynamics in Photoexcited MAPbBr3 by Infrared Spectroscopy
Source: J Phys Chem C Nanomater Interfaces. 2023 Nov 3;127(45):22097–104. doi: 10.1021/acs.jpcc.3c03668 (PMC10658633; doi:10.1021/acs.jpcc.3c03668)
Supplement: Supplementary file 1 — jp3c03668_si_001.pdf [file jp3c03668_si_001.pdf]

Supporting Information

Signatures of Polaron Dynamics in Photoexcited $MAPbBr_3$ by Infrared Spectroscopy

Valentina Carpenella[1], Claudia Fasolato[2], Diego Di Girolamo[3] Jessica Barichello[4], Fabio Matteocci[4], Caterina Petrillo[5], Danilo Dini[3] and Alessandro Nucara[6*]*

[1] Department of Sciences, University of Roma Tre, Via della Vasca Navale 84, 00146 Rome, Italy

[2] CNR-ISC, Istituto dei Sistemi Complessi, c/o Sapienza University of Rome, P.le A. Moro 5, 00185 Rome, Italy

[3] Department of Chemistry, Sapienza University of Rome, P.le A. Moro 5, 00185 Rome, Italy

[4] CHOSE, Department of Electronic Engineering, University of Rome Tor Vergata, Rome, 00133 Italy

[5] Department of Physics and Geology, University of Perugia, Via A. Pascoli, 06123, Perugia, Italy

[6] CNR-SPIN and Department of Physics, Sapienza University of Rome, Piazzale Aldo Moro 5, 00185 Rome, Italy

*Corresponding Authors: claudia.fasolato@cnr.it; alessandro.nucara@uniroma1.it

S1. Characterization of the films with Raman spectroscopy

All the Raman spectra reported in this section are collected with a Horiba HR Evolution micro-spectrometer employing 633 nm HeNe excitation laser operated in backscattering configuration. The Raman spectra of Sample A and Sample B are reported in Figure S1.

Assignation of the vibrational modes is provided in Table S1.

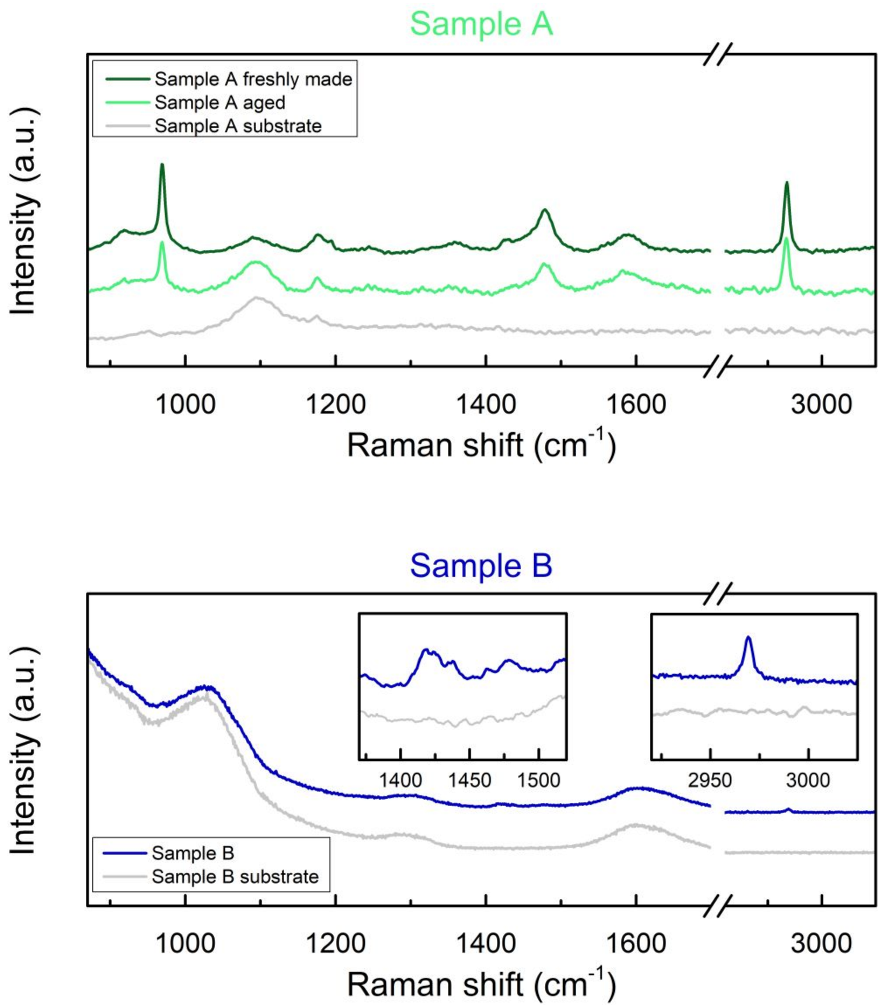

Figure S1. Top panel: Raman spectrum of Sample A acquired right after its synthesis (dark green) and after some months (green). In gray, the Raman spectrum of the layered glass-FTO-NiO-Al_2O_3-$PbBr_2$ substrate. Bottom panel: Raman spectrum of Sample B (blue). In gray, the Raman spectrum of the $SrTiO_3$ substrate. In the insets, magnification of the Sample B spectrum in the regions of the $MAPbBr_3$ Raman modes.

**Peak Position (cm^{-1})**	**Assignment**
916	MA rocking
969	C-N stretching
1428	CH_3 symm. bending
1478	NH_3^+ symm. bending
1590	NH_3^+ asymm. bending
2967	CH_3 asymm. stretching

Table S1. Summary of the observed $MAPbBr_3$ vibrational modes assigned from Ref. [*1*].

S2. UV-Vis absorption and Photoluminescence spectrum of Sample B and sample A

The UV-Vis spectrum was collected as already described in the main text, while the photoluminescence (PL) spectrum was collected on an optical table-mounted Raman setup. Excitation of the sample was achieved with a wavelength-tunable Melles-Griot Argon ion laser emitting a 488 nm laser line. The power was kept below 10 mW. The photoluminescence signal was collected in backscattering geometry using an Olympus BX62 microscope equipped with an

objective of 10x magnification. The beam was then analyzed with a Triax 320 single pass Czerny-Turner spectrometer by Horiba-Jobin Yvon, employing an 1800 grooves/mm diffraction grating and a Peltier-cooled charge-coupled device (CCD) detector. As already described by M. Hirasawa et al. [*2*], and schematically reported in the energy level diagram of Figure S2b, the two features in the absorbance spectrum can be associated with two possible transitions at the R point of the Brillouin zone when accounting for spin-orbit (SO) coupling.

The observed absorption edge is in good agreement with the value reported in the literature [3, 4] The PL spectrum, asymmetric in shape, was analyzed employing a two-peak convolution fit. This analysis shows that the PL profile is composed of a main peak centered at 2.28 eV (544 nm), and a broader, secondary peak at lower energies 2.26 eV (549 nm), whose origin can be ascribed to the presence of intragap energy levels.

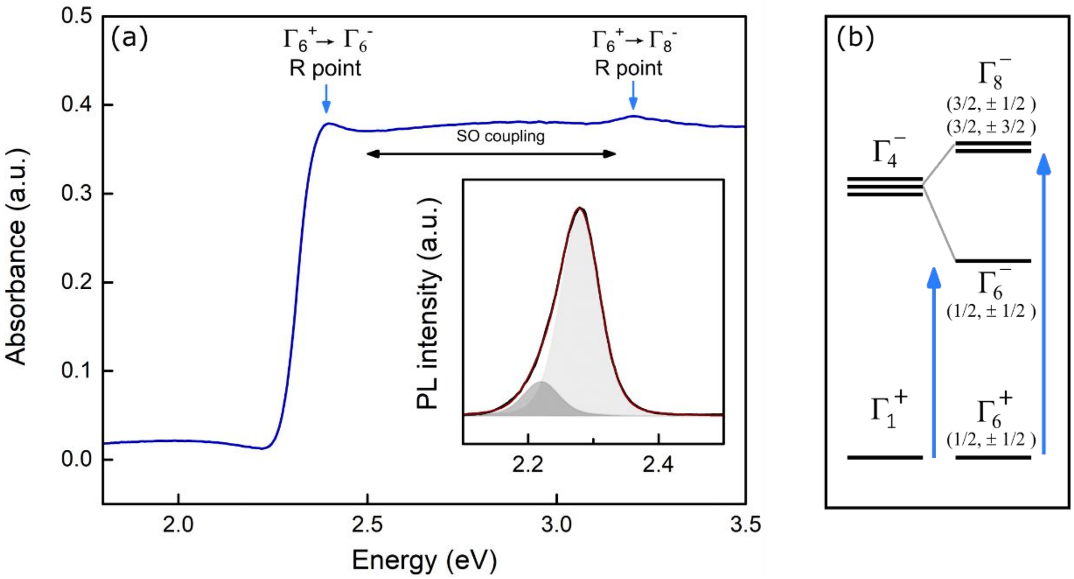

Figure S2. (a) Absorbance spectrum in the UV-Vis range of Sample B. The vertical blue arrows highlight the two electronic transitions of $MAPbBr_3$, in good agreement with the assignation reported in the literature [*2*]. This is schematically summarized in panel (b). In the inset of (a) the PL spectrum of Sample B collected with 488 nm excitation is shown.

The UV-Vis spectrum of sample A and that of the bare glass-FTO-NiO-Al_2O_3-$PbBr_2$ substrate is reported in Figure S3. The general downward trend with energy of the sample A spectrum can be qualitatively ascribed to the substrates behavior, as shown by the dashed grey line in Figure S3.

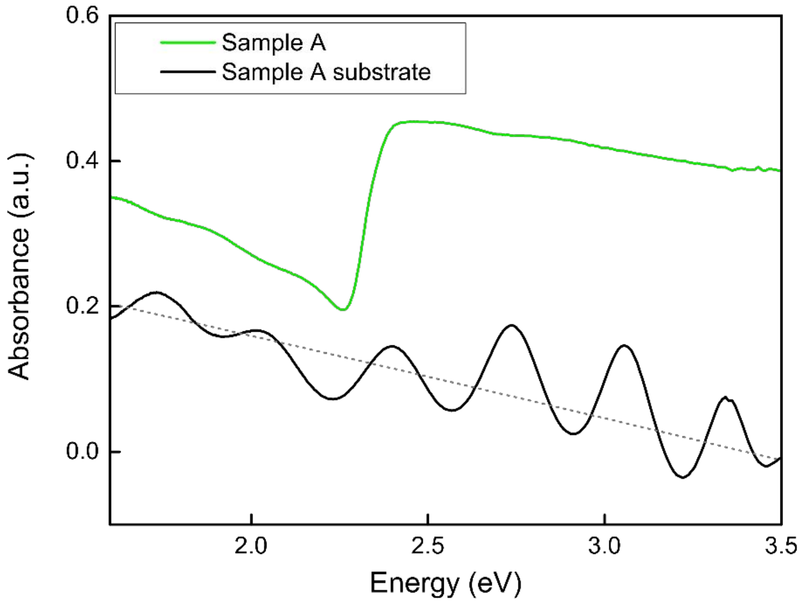

Figure S3. UV-Vis absorption spectrum of sample A (green) and of the sample A substrate (black). The dashed grey line is reported as a guide to the eyes to highlight the slope of the data. Interference fringes are visible on the spectrum of the substrate alone, due to its heterostructured composition.

S3. Estimation of the number of polaron

The temporal evolution of polaron population can be described by the following equation:

$$\frac{dn_p}{dt} = \beta - kn_p$$

in which β is the photoinduced polaron formation rate, and $\tau = 1/k$ is the characteristic decay time of the polaron population.

In the present experiment, in which each spectrum acquisition is about 20 sec long, we can reasonably assume that the number of detected polarons follows the asymptotic solution:

$$n_p \xrightarrow{t \to \infty} \beta\tau$$

S4. Polaron absorption coefficient

A third sample, prepared with the same protocol of sample B but with a precursor concentration of 0.8 M in the initial solution, was synthesized for the estimation of the polaron absorption coefficient. The thickness of this film sample was measured at 101 nm.

The absorption coefficient in the IR was estimated using the relation $\alpha_{pol} = (A_2 - A_1)/(d_2 - d_1)$, where A_2 and A_1 are the normalized polaronic absorbances of the two perovskite films deposited on STO of thicknesses 300 and 100 nm respectively, and the term d_2-d_1 is the difference between the thicknesses of the two films.

This calculation returns an absorption coefficient of α_{Pol} in the range of 10-100 cm^{-1}, where the variability depends on the efficiency in polaron creation by the optical laser pumping.

S5. Estimate of the number of incident photons from laser power

The number of photons absorbed by the sample as a function of the laser output power was estimated as follows:

$$n_{photons} = \frac{P}{h\nu}\frac{1}{S\,d}(1 - e^{-\alpha d})$$

being P is the optical output power of the laser diode (in Watt), S is the illuminated area (in cm^2) and d the film thickness. This quantity also accounts for $MAPbBr_3$ absorption coefficient α at 450 nm.

S6. Effects of temperature on the differential ΔA spectrum

For the differential absorbance data interpretation, we here employ a two-level system model, in which the two levels are excited by IR and external laser irradiation.

In this (semiclassical) model, only the ground state (G) and the first vibrational level (W) of a given molecular oscillator (e.g. the NH stretching mode at 3149 cm^{-1}) are considered. In presence of IR radiation and laser excitation, the population for the W and G levels vary with rates $\dot{n}_w$ and $\dot{n}_G$, respectively, in accordance with the kinetics equations:

$$\dot{n}_w = \delta - k_1 n_w - k_2 n_w + k_3 n_G \qquad (1)$$

$$\dot{n}_G = -(\dot{n}_w)$$

where:

δ is the rate of excitation of the vibrational state induced by indirect laser pumping;

k_1 is the spontaneous emission coefficient for radiative decay;

k_2 is the stimulated emission coefficient which includes the IR radiation density;

k_3 is the stimulated absorption coefficient which includes the IR radiation density;

In the limit of $k_1 \gg k$ (being $k = k_2 = k_3$) and $\delta = 0$ the steady state populations under IR excitation reduce to

$$\begin{cases} n_w \sim 0 \\ n_G \sim n_G^0 \end{cases}$$

Within the same approximation (δ=0) it is possible evaluate the percentage variation of the ground state population $\Delta n_G/n_G^0$ at different temperatures. This quantity is simply given by

$$\frac{\Delta n_G}{n_G^0} = -\frac{1}{\left(e^{\beta h\nu} + 1\right)}$$

and is proportional to the relative variation of absorption intensity.

If the influence of the laser is considered only as a thermal source rather than a promoter of the population of the W state, we can still use the approximation $\delta = 0$ and evaluate the value of the relative population of the ground state due to the increase in temperature induced by the laser. An increase of temperature ΔT upon light irradiation can be calculated by the expression $\Delta T = \frac{\alpha D}{\rho c}$, where α (cm^{-1}) is the absorption coefficient at 450 nm, D the energy density (or fluence in J cm^{-2}) of the laser at the spot of incidence with a given size, ρ the sample mass density (in g cm^{-3}) and c the specific heat (in J K^{-1} g^{-1}) [*6*]. However, the use of this relation lead to unphysical results in our case, as the poorness of the model does not account for the complex heterostructure of our sample. Thus, we measured the temperature of the film with a thermocouple as close as possible to the laser spot. just after 60 s of illumination. This method suffers for poor thermal contacts of the thermocouple and returns underestimated values of the film surface temperature. Therefore, we arbitrarily consider the temperature of the sample as twice and three times the value that was detected. The measured temperatures corresponding to different laser powers are reported in Figure S4a. It is to be noted that the estimation of the sample local temperature here reported does

not exceed $MAPbBr_3$ melting temperature (>450 K) [*7*]. As it can also be seen directly on the IR spectra, the features collected on the photoexcited films resembles the ones observed on the unperturbed one, meaning that the HOIP crystals did not undergo any substantial phase transition in the illumination conditions employed in this experiment. In Figure S4b, values of the relative ground state variation due to thermal effects and the experimental relative absorption detected upon laser irradiation are reported for a comparison. At the frequency of the NH bending mode, 1470 cm^{-1}, the observed decrease in absorption is compatible with the thermal effect alone, assuming that the local temperature reaches the values in Figure S4a. The result for the frequency of NH stretching (~3100 cm^{-1}) is drastically different, since between the theoretical estimate and the experimental values there are several orders of magnitude in favor of the latter. This result advises for the crucial role of the electronic photoexcitation, which affects the constant rate δ and the presence of which cannot be neglected in the case of high frequency molecular modes.

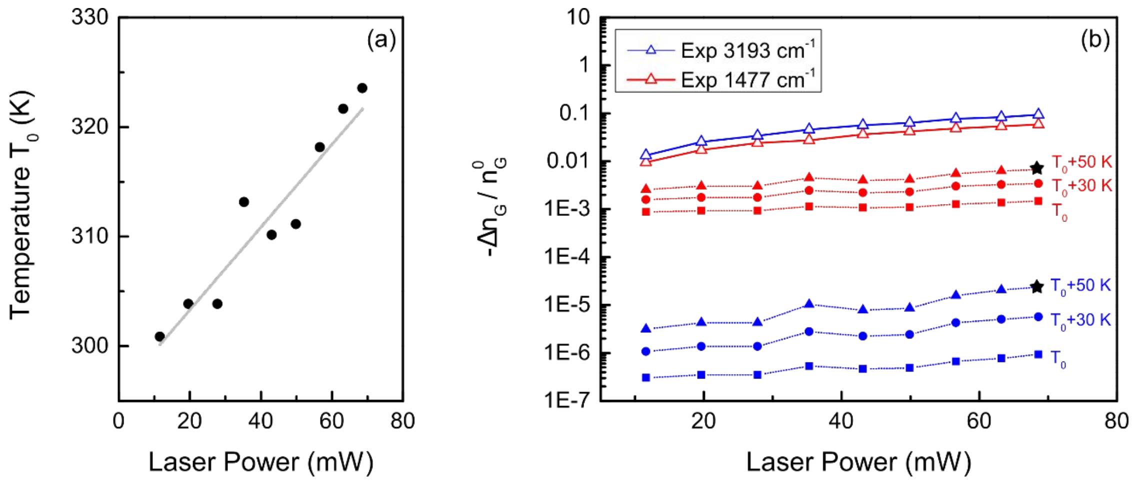

Figure S4. (a) Temperatures measured on the $MAPbBr_3$ film at different laser power illuminations. (b) Comparison between the percentage variation of the ground state population calculated at T (solid squares), T+30 K (solid circles) and T+50 K (solid triangles), and the variation of the absorbance spectrum obtained from experimental data $\Delta A/A_0$ (empty triangles, see main text) around 3149 cm^{-1} in blue, and 1477 cm^{-1} in red. The black stars point to the values of $\Delta n_G/n_G^0$ close to the melting temperature of $MAPbBr_3$.

S7. Differential absorbance of Sample B at 3000 cm^{-1}

The differential absorbance of Sample B is reported in figure S5 at different laser output powers.

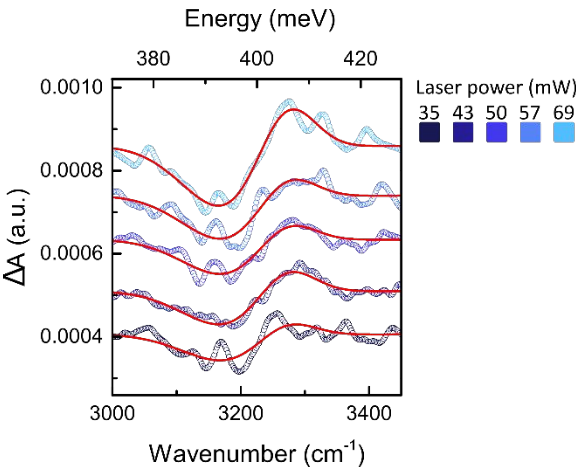

Figure S5. Differential absorbance ΔA of Sample B in the NH stretching mode region, at different laser output power. The red line following the data is shown as a guide for the eye.

S8. Baseline subtraction of the differential absorbance (ΔA) spectra

A spline baseline has been subtracted to the differential spectra of the $MAPbBr_3$ heterostructure (Sample A) shown in the main text. The shape of the baseline is shown in Figure S6 (red trace).

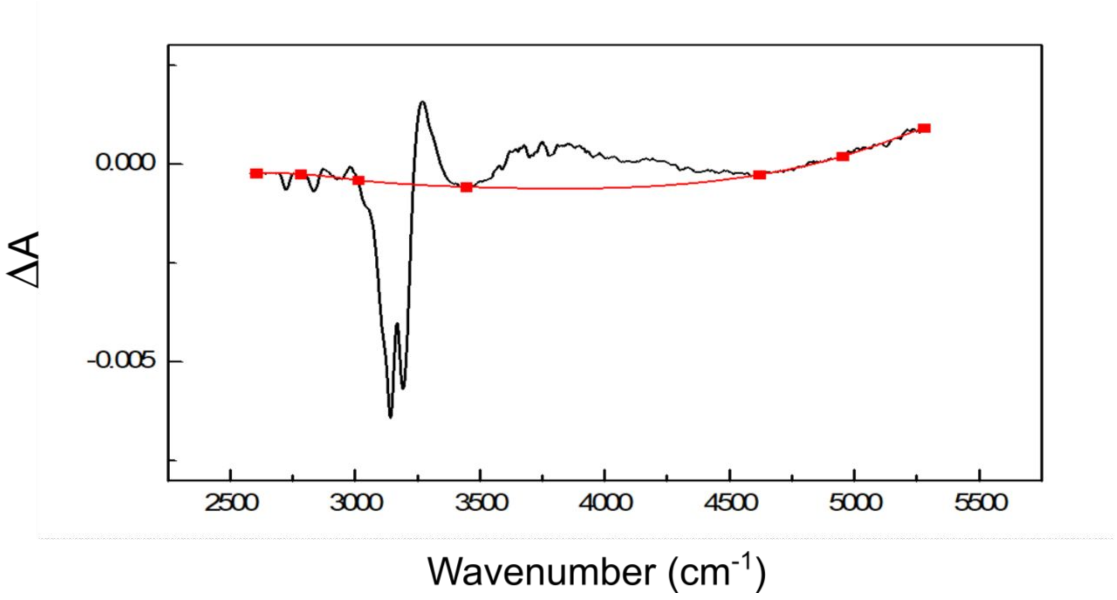

Figure S6. Baseline spline subtraction on the spectra corresponding to an optical laser output power of 69 mW.

S9. Possible orientations of the methylammonium cation in the distorted cage

The orientations of MA cation in the cage are shown in Figure S7, in accordance with the calculations by J. H. Lee et al. for $MAPbI_3$ in the tetragonal symmetry. Figure S7 is adapted from ref. [*8*]

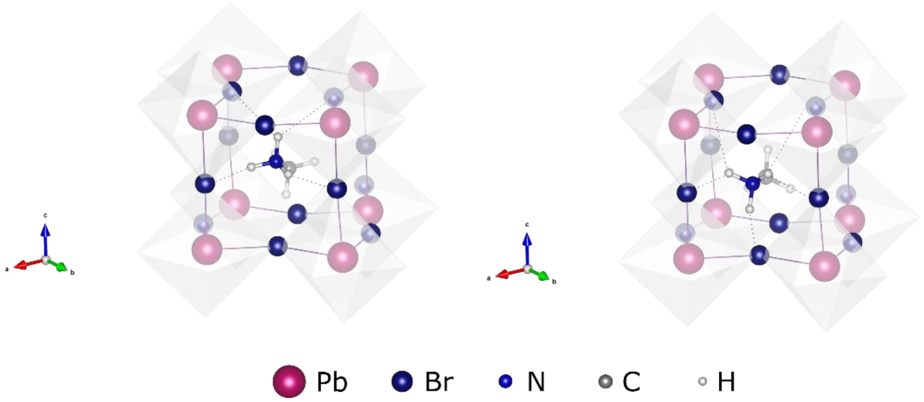

Figure S7 Graphic representation of the MA orientations in the $MAPbI_3$ tetragonal cell. The figure is adapted from Figure 7 in J. H. Lee et al. [*8*] (CC BY Creative Common licence).

References

[*1*] Niemann, R. G., Kontos, A. G., Palles, D., Kamitsos, E. I., Kaltzoglou, A., Brivio, F., ... & Cameron, P. J. Halogen effects on ordering and bonding of $CH_3NH_3^+$ in $CH_3NH_3PbX_3$ (X= Cl, Br, I) hybrid perovskites: a vibrational spectroscopic study. *The Journal of Physical Chemistry C* **2016**, 120(5), 2509-2519.

[*2*] Hirasawa, M., Ishihara, T., & Goto, T. Exciton features in 0-, 2-, and 3-dimensional networks of $[PbI_6]^{4-}$ octahedra. *Journal of the Physical Society of Japan* **1994**, 63(10), 3870-3879.

[*3*] Mannino, G., Deretzis, I., Smecca, E., La Magna, A., Alberti, A., Ceratti, D., & Cahen, D. Temperature-dependent optical band gap in $CsPbBr_3$, $MAPbBr_3$, and $FAPbBr_3$ single crystals. *The journal of physical chemistry letters* **2020**, 11(7), 2490-2496.

[*4*] Chen, C., Hu, X., Lu, W., Chang, S., Shi, L., Li, L., ... & Han, J. B. Elucidating the phase transitions and temperature-dependent photoluminescence of $MAPbBr_3$ single crystal. *Journal of Physics D: Applied Physics* **2018**, 51(4), 045105.

[5] Wehrenfennig, C., Liu, M., Snaith, H. J., Johnston, M. B., & Herz, L. M. Charge-carrier dynamics in vapour-deposited films of the organolead halide perovskite $CH_3NH_3PbI_{3-x}Cl_x$. *Energy & Environmental Science* **2014**, 7(7), 2269-2275.

[*6*] De Giorgi, M. L., Lippolis, T., Jamaludin, N. F., Soci, C., Bruno, A., & Anni, M. Origin of amplified spontaneous emission degradation in mapbbr3 thin films under nanosecond-uv laser irradiation. *The Journal of Physical Chemistry C* **2020**, 124(19), 10696-10704.

[*7*] Brunetti, B., Cavallo, C., Ciccioli, A., Gigli, G., & Latini, A. On the thermal and thermodynamic (in) stability of methylammonium lead halide perovskites. *Scientific reports* **2016**, 6(1), 1-10.

[*8*] Lee, J. H., Lee, J. H., Kong, E. H., & Jang, H. M. The nature of hydrogen-bonding interaction in the prototypic hybrid halide perovskite, tetragonal $CH_3NH_3PbI_3$. *Scientific reports* **2016**, 6(1), 21687.
